# Relationships between computer-extracted mammographic texture pattern features and *BRCA1/2*mutation status: a cross-sectional study

**DOI:** 10.1186/s13058-014-0424-8

**Published:** 2014-08-23

**Authors:** Gretchen L Gierach, Hui Li, Jennifer T Loud, Mark H Greene, Catherine K Chow, Li Lan, Sheila A Prindiville, Jennifer Eng-Wong, Peter W Soballe, Claudia Giambartolomei, Phuong L Mai, Claudia E Galbo, Kathryn Nichols, Kathleen A Calzone, Olufunmilayo I Olopade, Mitchell H Gail, Maryellen L Giger

**Affiliations:** 1 Hormonal and Reproductive Epidemiology Branch, Division of Cancer Epidemiology and Genetics, National Cancer Institute, National Institutes of Health, 9609 Medical Center Drive, Rm. 7-E108, Bethesda, 20892-9774 MD USA; 2 Department of Radiology, University of Chicago, Chicago, IL USA; 3 Clinical Genetics Branch, Division of Cancer Epidemiology and Genetics, National Cancer Institute, National Institutes of Health, Bethesda, MD USA; 4 Washington Radiology Associates, Potomac, MD USA; 5 Coordinating Center for Clinical Trials, Office of the Director, National Cancer Institute, National Institutes of Health, Bethesda, Maryland USA; 6 Genentech, South San Francisco, CA USA; 7 Department of Surgery, Uniformed Services University of the Health Sciences, Bethesda, MD USA; 8 Genetics Institute, University College London, London, UK; 9 Department of Radiological Sciences, Uniformed Services University of the Health Sciences, Bethesda, Maryland USA; 10 Department of Radiology, Walter Reed National Military Medical Center, Bethesda, Maryland USA; 11 Westat, Inc., Rockville, Maryland USA; 12 Genetics Branch, Center for Cancer Research, National Cancer Institute, National Institutes of Health, Bethesda, Maryland USA; 13 Center for Clinical Cancer Genetics, Department of Medicine, University of Chicago, Chicago, IL USA; 14 Biostatistics Branch, Division of Cancer Epidemiology and Genetics, National Cancer Institute, National Institutes of Health, Bethesda, MD USA

## Abstract

**Introduction:**

Mammographic density is similar among women at risk of either sporadic or *BRCA1/2*-related breast cancer. It has been suggested that digitized mammographic images contain computer-extractable information within the parenchymal pattern, which may contribute to distinguishing between *BRCA1/2* mutation carriers and non-carriers.

**Methods:**

We compared mammographic texture pattern features in digitized mammograms from women with deleterious *BRCA1/2* mutations (*n* = 137) versus non-carriers (*n* = 100). Subjects were stratified into training (107 carriers, 70 non-carriers) and testing (30 carriers, 30 non-carriers) datasets. Masked to mutation status, texture features were extracted from a retro-areolar region-of-interest in each subject’s digitized mammogram. Stepwise linear regression analysis of the training dataset identified variables to be included in a radiographic texture analysis (RTA) classifier model aimed at distinguishing *BRCA1/2* carriers from non-carriers. The selected features were combined using a Bayesian Artificial Neural Network (BANN) algorithm, which produced a probability score rating the likelihood of each subject’s belonging to the mutation-positive group. These probability scores were evaluated in the independent testing dataset to determine whether their distribution differed between *BRCA1/2* mutation carriers and non-carriers. A receiver operating characteristic analysis was performed to estimate the model’s discriminatory capacity.

**Results:**

In the testing dataset, a one standard deviation (SD) increase in the probability score from the BANN-trained classifier was associated with a two-fold increase in the odds of predicting *BRCA1/2* mutation status: unadjusted odds ratio (OR) = 2.00, 95% confidence interval (CI): 1.59, 2.51, *P* = 0.02; age-adjusted OR = 1.93, 95% CI: 1.53, 2.42, *P* = 0.03. Additional adjustment for percent mammographic density did little to change the OR. The area under the curve for the BANN-trained classifier to distinguish between *BRCA1/2* mutation carriers and non-carriers was 0.68 for features alone and 0.72 for the features plus percent mammographic density.

**Conclusions:**

Our findings suggest that, unlike percent mammographic density, computer-extracted mammographic texture pattern features are associated with carrying *BRCA1/2* mutations. Although still at an early stage, our novel RTA classifier has potential for improving mammographic image interpretation by permitting real-time risk stratification among women undergoing screening mammography.

**Electronic supplementary material:**

The online version of this article (doi:10.1186/s13058-014-0424-8) contains supplementary material, which is available to authorized users.

## Introduction

Epidemiologic studies have consistently demonstrated that elevated mammographic density is a strong and independent risk factor for sporadic breast cancer, conferring relative risks of 4- to 5-fold when comparing women with high versus low mammographic density [1]. Although mammographic density has a strong heritable component [2]-[10], it is currently being debated as to whether mammographic density is associated with hereditary breast cancer risk [11],[12]. Up to half of all hereditary breast cancer cases can be attributed to autosomal dominant mutations in two genes, *BRCA1* and *BRCA2*[13]. Among women with *BRCA1/2* mutations, nearly 50% may be expected to develop breast cancer by age 50 years [13]. The ability to identify high-risk patients through analysis of mammographic images could have clinically significant implications for breast cancer screening and prevention strategies.

Utilizing a computer-assisted method to characterize percent mammographic density (PMD), we have previously reported that mammographic density is not associated with *BRCA1/2* mutation status [14], a finding consistent with those from prior studies [12],[15]-[18]. In contrast, Huo *et al*. and Li *et al*. used computerized radiographic texture analysis of a retro-areolar region-of-interest (ROI) to distinguish between mutation carriers and low-risk women; mutation carriers had a breast parenchymal texture pattern that was characterized as being coarse with low contrast [19],[20].

Radiographic texture analysis (RTA) has long been utilized in medical imaging research, but investigators have taken different approaches when using texture analysis of mammographic images [19]-[30]. Broadly, the extracted mammographic features are described as gray-level magnitude-based features, which describe variation of gray-value intensities and ignore spatial relationships (for example*,* percent density), and texture-based features, which characterize the higher-order statistics of the spatial radiographic patterns.

Multiple investigators have evaluated whether texture-based features capture a component of risk beyond that of mammographic density [19],[22]-[26],[31],[32], but only Huo *et al*. and Li *et al*. have suggested that this method might accurately classify subjects according to *BRCA1/2* mutation status [19],[20]. These findings, though promising, were based on the analysis of 30 *BRCA1/2* mutation carriers. This study represents replication and validation of their results in a larger, independent dataset.

## Methods

### Study populations and data collection

The study populations have been described previously [14]. Briefly, the NCI Clinical Genetics Branch Breast Imaging Study evaluated breast cancer screening modalities in women who were at high genetic risk of breast cancer. From 2001 to 2007, 200 women were enrolled in this study, including 170 women with proven deleterious *BRCA1/2* mutations and 30 proven mutation-negative women from the same families. Participants were seen at the NIH Clinical Center (NCI Protocol #01-C-0009; NCT-00012415) and underwent a physical examination, nipple fluid aspiration, breast duct lavage, standard clinical four-view screening mammogram and breast magnetic resonance imaging (MRI), which were reviewed by the study radiologist (CKC). See prior reports for additional details related to study design [33],[34]. The NCI Institutional Review Board (IRB) approved the study, and all participants provided informed consent.

The NCI/National Naval Medical Center (NNMC) Susceptibility to Breast Cancer Study was a cross-sectional study of the association between mammographic density and genes involved in estrogen metabolism. From 2000 to 2006, 219 women with a documented personal history of breast cancer and 488 controls were enrolled. Participants were enrolled from the patient population at the NNMC and other referring institutions and the NIH Clinical Center (NNMC Protocol #NNMC.2000.0010; NCI Protocol #00-C-0079; NCT-00004565). Mammograms obtained within the year prior to enrollment were reviewed by two study radiologists (CKC and CEG). Study participants did not undergo *BRCA1/2* mutation testing. Five-year Gail assessment [35] and Pedigree assessment tool (PAT) [36] scores were calculated for all controls. The PAT is a point-scoring system that uses family cancer history to identify women who are at high risk of hereditary breast cancer (that is, >10% risk of being a *BRCA1/2* mutation carrier) [36]-[38]. A PAT score ≥8.0 has been associated with 100% sensitivity and 93% specificity for detecting mutation carriers, and a PAT score <8.0 has been associated with a negative predictive value of 100% [36]. For the current study, control subjects with low scores by both models were classified as having low risk of breast cancer; they were highly unlikely to be *BRCA1/2* mutation carriers. The IRBs of the NNMC and NCI approved this study, and all participants provided written informed consent.

Participants from both studies completed self-administered questionnaires which captured demographic characteristics, current weight and height, medical and reproductive history, and personal and familial history of cancer. Questionnaire items were compared between studies, and common response categories were combined in order to create a harmonized analytic database.

### Analytic sample

**Figure 1:**
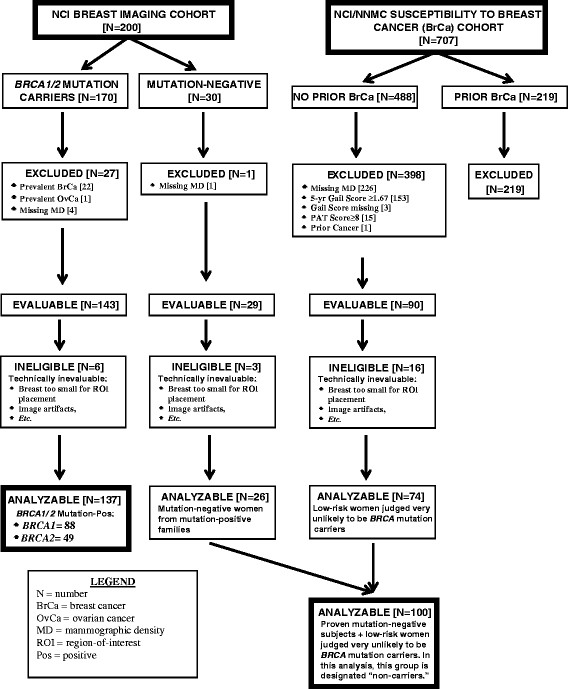
**Flow diagram depicting the eligibility criteria used to derive the analytic sample of** ***BRCA1/2*** **mutation carriers and non-carriers.** PAT, Pedigree assessment tool.

A flow diagram of the criteria utilized to derive the analytic sample of *BRCA1/2* mutation carriers and non-carriers is depicted in Figure 1.

#### The NCI Clinical Genetics Branch’s Breast Imaging Study

After excluding 22 women with prevalent breast cancer (11 *BRCA1* carriers, 11 *BRCA2* carriers), one *BRCA1* carrier with prevalent ovarian cancer, five women with missing mammographic density readings (three *BRCA1* carriers, one *BRCA2* carrier, and one non-carrier whose mammograms were given to the patients for care in their home communities prior to being digitized), the final study population included 143 mutation carrier and 29 non-carrier women (the latter from mutation-positive families) eligible for analysis. Of these, images from six mutation carrier and three non-carrier women were deemed ineligible for analysis of computer-extracted texture features for various reasons (for example, breast area too small for ROI placement, image artifacts, et cetera), resulting in a total of 137 mutation carriers (88 *BRCA1*- and 49 *BRCA2*-positive) and 26 non-carriers in our analytic sample.

#### The NCI/NNMC Susceptibility to Breast Cancer Study

For the purposes of this report, the analytic sample was restricted to controls with available mammographic density readings, who were determined to be at low-to-average breast cancer risk. After excluding controls with missing density readings (n = 226), 262 potentially eligible women remained. Of these, 153 women had a 5-year Gail score ≥1.67, three women were missing Gail scores, 15 women had PAT scores ≥8, and one woman had a personal history of skin cancer, type unspecified; these 172 women were excluded, resulting in 90 non-carriers eligible for analysis. Of these, images from 16 women were deemed ineligible for analysis of computer-extracted texture features for the reasons described above and were excluded, yielding 74 women at low-to-average risk of breast cancer for our analytic sample. Medians (ranges) for their maternal PAT, paternal PAT and 5-year Gail scores were 0 (0, 7), 0 (0, 5), and 1.2 (0.3, 1.6), respectively. Given the rarity of *BRCA1/2* mutations in the general population, and the low PAT scores, these 74 women were assumed to be mutation-negative. For the sake of simplicity, combining these women with the 26 known mutation-negative subjects from the Breast Imaging Study, we use the term “non-carriers” in this report to describe these 100 women.

### Assessment of mammographic density

Analog mammographic films from both studies were digitized at 0.095 mm (267 dots per inch) in pixel size and 8-bit quantization in gray level. The details of the digitization process have been described previously [14]. Participants from both studies had standardized, quantitative calculations of PMD measured in digitized craniocaudal views by the same experienced study mammographer (CKC), using an interactive computerized thresholding method developed at the NIH Clinical Center (MEDx™ version 3.44, Medical Numerics, Germantown, MD, USA). We have previously reported that the intra-observer agreement for PMD assessed in 100 paired sets using MEDx was 0.89 [14]. In addition, we found that Cumulus™ measures of PMD were strongly and positively correlated with those assessed by MEDx (*r* = 0.84, *P* <0.0001) [14].

### Computerized assessment of mammographic parenchymal patterns

**Figure 2:**
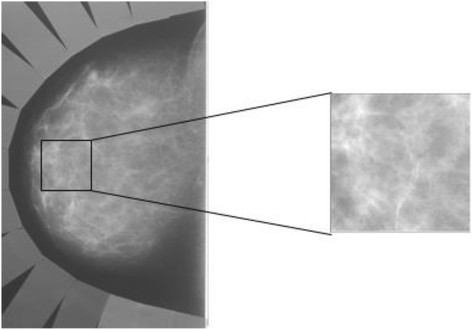
**A sample region-of-interest (ROI) selected from central breast region behind the nipple on a digitized mammogram.**

Regions-of-interest (ROIs) measuring 256 by 256 pixels were manually selected by the same investigator (LL) without knowledge of *BRCA1/2* mutation status, from the central breast region behind the nipple on digitized craniocaudal projections (Figure 2). Detailed explanations of the effects of ROI extraction, ROI size, and ROI location on RTA have been reported elsewhere [20]. These ROIs were used in the subsequent analytic step to extract and characterize the gray-level magnitude-based and parenchymal texture-based features of the digitized mammograms.

### Radiographic texture analysis (RTA) of computer-extracted features

The detailed descriptions of the 38 computer-extracted parenchymal texture features (mathematical descriptors used in the RTA) have been reported previously [19],[20],[27]-[30],[39]-[41]; their feature numbers, names and definitions are summarized in Additional file 1: Table S1. For ease of interpretation, gray-level magnitude (M)-based features were assigned alpha-numeric descriptors ranging from M1 to M9, and texture (T)-based features were assigned descriptors ranging from T1 to T29. We assessed the internal reliability of the reader’s ROI placement by randomly submitting a masked set of 91 mammograms (Susceptibility to Breast Cancer Study (n = 27); Breast Imaging Study (n = 64)) for re-selection of the ROIs and re-analysis by the RTA algorithms. The intraclass correlation coefficient (ICC) was calculated to assess the intra-observer reliability of the RTA features following manual re-selection of the ROIs.

### Statistical analyses

#### Selection of participants for the training and testing datasets

After exclusions, 237 subjects were eligible for analysis: 137 *BRCA1/2* carriers, 100 non-carriers. We divided these women into a training set used to develop discrimination models to distinguish carriers from non-carriers, and a testing set used to evaluate how well the discrimination model distinguished carriers from non-carriers. From the 100 non-carriers, 6 were randomly selected from each non-carrier quintile of age, for a total of 30 non-carriers, to comprise the testing set. Likewise, from the 137 mutation carriers, 6 women were randomly selected from each carrier quintile of age, yielding 30 carriers for the testing set. The remaining 177 women comprised the training dataset. Baseline characteristics were compared between *BRCA1/2* mutation carriers and non-carriers within the training and testing datasets using the two-sample *t*-test for independent samples. We assumed equal variances for continuous measures, and used the chi-square test for discrete measures.

#### Stepwise feature selection using linear discriminant analysis

Utilizing the 177 subjects in the training dataset, we employed stepwise feature selection using linear discriminant analysis, in which RTA features were reiteratively added and removed from the group of selected features based on a feature selection criterion, that is, the Wilks’ lambda [42],[43]. In each iteration step, linear discriminant analysis was used to calculate the discriminant scores, which were then used to compute the Wilks’ lambda. The *F*-statistic was applied to determine whether a particular feature contributed significantly (*P*-value <0.05) to the performance of the linear discriminant analysis in each step. Details of stepwise feature selection using linear discriminant analysis are described in Additional file 2. The stepwise feature selection was performed 177 times, by leaving out one woman from the training set each time. To be included as a classifier for distinguishing carriers from non-carriers, a feature had to be selected in at least half of these 177 analyses.

#### Merging of computer-extracted features

The RTA features selected in the linear discriminant analysis were combined using a Bayesian artificial neural network (BANN) algorithm (Additional file 2). The output from BANN was converted to an estimate (probability score) of the likelihood of being within the *BRCA1/2* mutation carrier group. These probability scores were evaluated for their capacity to serve as an image-based marker of risk in the independent testing data set by assessing whether their distribution differed between *BRCA1/2* mutation carriers and non-carriers. In order to assess how mammographic density might influence the discrimination performance, we also developed (training data) and tested (testing data) a modified BANN classifier in which percent mammographic density was forced to be included along with the same selected RTA features. Both the linear discriminant analysis and the BANN algorithm were completed in MatLab™ (The MathWorks, Inc. Natick, MA, USA).

#### Performance evaluation and related statistical analyses

Spearman’s rank correlation coefficient was used to describe the relationships between the selected computerized texture features with PMD, age and each other. The ability of the BANN-trained classifier to distinguish between *BRCA1/2* mutation carriers and non-carriers was evaluated in the testing dataset using several approaches. We evaluated the relation between the BANN-trained classifier output and *BRCA1/2* mutation status in univariate and multivariable logistic regression analysis, first adjusted for age as a continuous variable, and then adjusted for age and PMD. For comparison purposes, we evaluated the relationships between (a) PMD alone, and (b) the modified BANN-trained classifier, which included PMD with *BRCA1/2* mutation status in both univariate and multivariable logistic regression analysis adjusted for age. In sensitivity analyses, we additionally adjusted for baseline characteristics that differed by mutation status.

Because carriers were on average approximately 10 years younger than non-carriers [14], we also performed age-matched sensitivity analysis in the testing data. First, we applied the BANN-trained classifier from the original training dataset to testing datasets restricted to pairs of *BRCA1/2* mutation carriers and non-carriers who were randomly selected and matched on age within ±3 years (that is, 19 mutation carriers, 19 non-carriers) and ±1 year (that is, 17 mutation carriers, 17 non-carriers). Within the age-matched testing datasets, the Wilcoxon signed rank test was used to examine the mean paired difference in the BANN probability score between carriers and non-carriers. We performed a similar paired difference analysis of BANN probability scores based on the selected features and PMD. In an additional sensitivity analysis, we removed women older than age 55 years from both the training and testing datasets, and repeated the analysis conducted with the combined dataset.

The utility of the computer-extracted RTA features, as well as the output from BANN in the task of differentiating the two groups, was also evaluated by using receiver operating characteristic (ROC) analysis [44],[45]. The area under the fitted ROC curve (AUC) was used to evaluate the inherent discriminant capacity of the decision variable. The AUC measures the probability that a randomly-selected carrier will have a greater probability score than a randomly-selected non-carrier. The ROCKIT™ software package (ROCKIT, version 1.1b) [46] was used to evaluate the statistical significance of the difference between two AUC values (that is, the AUC from the BANN-trained classifier was compared with the AUC from PMD alone) [47]. We used two methods to obtain age-adjusted estimates of the AUC values explained by the BANN-trained classifier. In the first method, we restricted our test-set to pairs age-matched within ±3 and ±1 years, as defined above. In an alternate approach, we computed individual AUCs within age strata, in which the testing dataset was divided into three age strata: 25 to <35 years, 35 to <45 years, and 45 to 55 years. The AUCs were computed within each age stratum, and then were averaged to yield the AUC across the age strata. Except where noted above, analyses were completed using SAS statistical software (SAS 9.2 software, SAS Institute Inc., Cary, NC, USA). Probability values <0.05 were considered to be statistically significant. All tests of statistical significance were two-tailed.

## Results

### Distribution of patient characteristics in the training and testing datasets

The baseline characteristics of *BRCA1/2* mutation carriers and non-carriers stratified by training and testing datasets are shown in Table 1. Compared with non-carriers, the *BRCA1/2* mutation carriers were statistically significantly younger, more likely to be white, nulliparous or to have a later age at first birth, and to have undergone surgical menopause. As previously reported, age-adjusted mean PMD did not differ between *BRCA1/2* carriers and non-carriers [14].

**Table 1:** **Baseline characteristics of** ***BRCA1/2*** **mutation carriers and non-carriers according to the training and testing datasets**

	Training dataset (n = 177)					Testing dataset (n = 60)						
Variable	Non-carriers (n = 70)		Unaffected*BRCA1/2*carriers (n = 107)		*P*-value for mutation carriers versus non-carriers	Non-carriers (n = 30)		Unaffected*BRCA1/2*carriers (n = 30)		*P*-value for mutation carriers versus non-carriers	*P*-value^2^	*P*-value^3^
	Mean (SD)	Range	Mean (SD)	Range	*P*-value from*t*-test	Mean (SD)	Range	Mean (SD)	Range	*P*-value from*t*-test	*P*-value from*t*-test	
Age, years	48.8 (9.6)	25, 79	37.7 (8.5)	22, 55	**<0.0001**	47.2 (10.1)	25, 74	37.8 (8.9)	25, 55	**0.0003**	0.44	0.95
Body mass index^1^	26.2 (5.7)	18.0, 45.5	25.5 (5.4)	17.9, 48.2	0.41	27.3 (6.7)	18.3, 49.5	25.9 (5.9)	19.5, 40.0	0.37	0.38	0.74
Percent mammographic density, unadjusted	32.2 (15.2)	3.5, 76.3	37.6 (14.7)	2.8, 70.8	**0.02** ^**4**^	30.7 (14.7)	2.0, 53.7	35.5 (14.2)	4.8, 68.4	0.21^4^	0.66	0.47
	**Number**	**%** ^*^	**Number**	**%** ^*^	***P*** **-value from chi square** ^**5**^	**Number**	**%** ^*^	**Number**	**%** ^*^	***P*** **-value from chi square** ^**5**^	***P*** **-value from chi square** ^**5**^	
White, non-Hispanic	63	90.0	107	100.0	**0.001**	25	83.3	30	100.0	0.05	0.35	1.00
College graduate	52	74.3	83	77.6	0.62	21	70.0	22	73.3	0.77	0.66	0.63
Ever smoked	17	24.3	35	32.7	0.23	13	43.3	8	26.7	0.18	0.06	0.53
Age at menarche, years					0.78					0.87	0.96	0.40
<12	10	14.5	13	12.3		5	16.7	6	20.0			
12 to 13	42	60.9	70	66.0		18	60.0	16	53.3			
≥14	17	24.6	23	21.7		7	23.3	8	26.7			
Missing	1		1									
Parous	52	74.3	61	57.0	**0.02**	24	80.0	13	43.3	**0.004**	0.54	0.18
Age at first birth, years					0.09					**0.02**	0.27	0.62
<30	36	51.4	41	38.3		19	63.3	10	33.3			
≥30 or nulliparous	34	48.6	66	61.7		11	36.7	20	66.7			
Ever used oral contraceptives	51	72.9	96	89.7	**0.004**	24	80.0	26	86.7	0.49	0.45	0.64
Menopausal status					**<0.0001**					0.08	0.59	0.83
Premenopausal	44	64.7	60	56.1		16	57.1	19	63.3			
Postmenopausal, natural	4	5.9	6	5.6		4	14.3	2	6.7			
Postmenopausal, surgical	9	13.2	40	37.4		4	14.3	9	30.0			
Postmenopausal, unknown	11	16.2	1	0.9		4	14.3	0	0.0			
Missing	2		0			2		0				
Menopausal hormone therapy					0.97					0.31	0.95	0.30
Never	48	68.6	74	69.2		20	66.7	25	83.3			
Former	10	14.3	16	15.0		5	16.7	2	6.7			
Current	12	17.1	17	15.9		5	16.7	3	10.0			
Breast biopsy prior to enrollment	21	30.0	26	24.3	0.40	5	16.7	7	23.3	0.52	0.16	0.91

*Missing values were excluded from percentage calculations. ^1^Weight (kg)/height^2^ (m^2^). ^2^*P*-value comparing non-carriers in the training set to those in the testing set. ^3^*P*-value comparing *BRCA1/2* carriers in the training set to those in the testing set. ^4^As previously reported [14], age-adjustment attenuatated the mean differences in percent mammographic density between carriers and non-carriers (age-adjusted *P*-value: training dataset = 0.79; testing dataset = 0.87). ^5^*P*-value from chi square test or Fisher’s exact test when appropriate. P-values <0.05 are shown in bold font.

Because women were randomly selected from age quintiles within each risk group for the training and testing datasets, the age distribution of non-carriers in the training set (n = 70) was similar to that of the testing set (n = 30) (*P* = 0.44). Likewise, the age distribution of the carriers in the training set (n = 107) was similar to those in the testing set (n = 30) (*P* = 0.95). The distributions of PMD within the risk groups were also similar between training and testing sets (non-carriers: *P* = 0.66; *BRCA1/2* carriers: *P* = 0.47). There were no statistically significant differences in body mass index (BMI) between the risk groups or between the training and testing datasets.

### Descriptive characteristics of selected computer-extracted features

**Figure 3:**
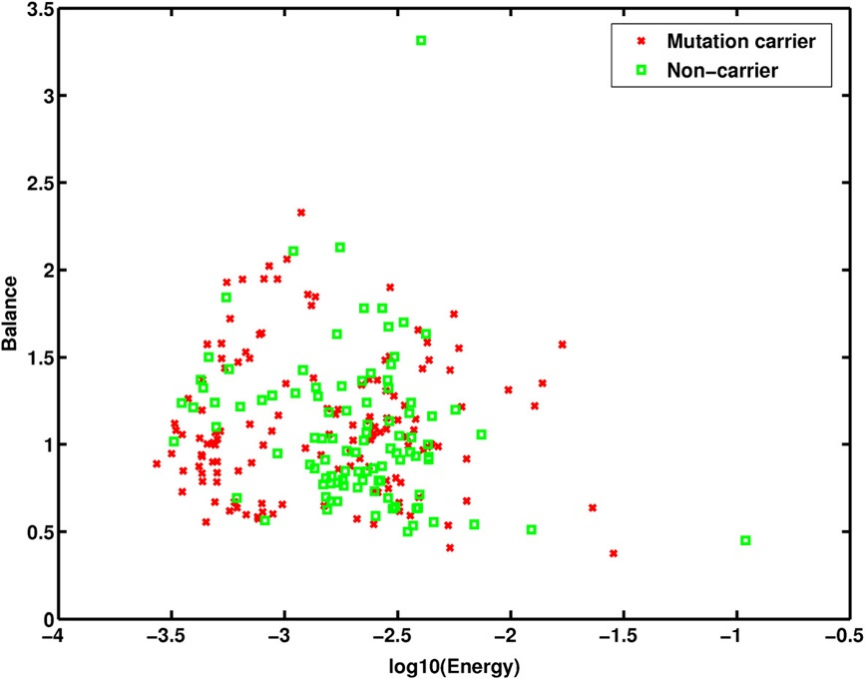
**Scatterplot of the computer-extracted parenchymal features of Energy and Balance for** ***BRCA1/2*** **mutation carriers and non-carriers.** Energy, a texture-based feature, was identified as distinguishing between carriers and non-carriers; Balance, a gray-level magnitude-based feature, was selected in age-matched analyses. Compared with non-carriers, mutation carriers tended to have a parenchymal texture with low Energy.

The ICCs between duplicate measurements of the 38 computer-extracted RTA features for the 91 women with repeated readings ranged from 0.79 to 0.99, documenting high reliability of ROI selection and analysis (Additional file 1: Table S1). Additional file 1: Figure S1 shows the number of times that each feature was selected in the 177 leave-one-case-out feature selection analyses of the training data. Of the 9 gray-level magnitude- and 29 texture-based computerized features explored using the training dataset, two gray-level magnitude- (that is, M1: AVE; M2: MinCDF) and two texture-based features (that is, T1: Energy; T2: MaxF (COOC) were selected more than half the time, and were therefore included in subsequent BANN models. A third gray-level feature, “Balance”, was selected in sensitivity analyses in which the training dataset was truncated at the upper age-limit of mutation carriers. The distribution of values for the selected features of Energy and Balance are shown in the scatter plot in Figure 3. This plot demonstrates that the parenchymal texture features of mutation carriers tend to have low Energy, that is, they are less homogenous, with a coarse pattern.

Table 2 presents descriptive information related to the selected features. On average, mutation carriers tended to have lower values for the gray-level magnitude-based features, and texture-based features were less homogeneous as compared with the non-carriers. With regard to the three selected gray-level magnitude-based features, the feature characterizing the average gray value within the ROI (“AVE”) was positively correlated with PMD (*r* = 0.31, *P* <0.0001), whereas the Balance feature was inversely correlated with PMD (*r* = -0.32, *P* <0.0001). A weak inverse correlation was observed between the MinCDF feature (that is, the gray value corresponding to the 5% region cutoff on the cumulative density function) and PMD (*r* = -0.13, *P* = 0.04); MinCDF was positively correlated with age (*r* = 0.23, *P* = 0.0005). Modest statistically significant inverse correlations were observed between PMD and both of the selected texture-based features, Energy and MaxF (COOC), which are measures of image homogeneity (Energy: *r* = -0.30, *P* < 0.0001; MaxF (COOC): *r* = -0.24, *P* = 0.0002). These selected texture-based features were positively correlated with age.

**Table 2:** **Descriptive characteristics of selected computer-extracted features**

			Correlation with percent mammographic density (n = 237)		Correlation with age (n = 237)		Non-carriers (n = 100)		Unaffected***BRCA1/2***carriers (n = 137)	
Feature type and number	Feature^1^	Definition	r*	***P***-value	r*	***P***-value	Mean (SD)	Range	Mean (SD)	Range
**Gray-level magnitude-based features:**										
M1	AVE	Average gray value within ROI; higher values correspond to denser region	**0.31**	**<0.0001**	0.01	0.89	139.6 (26.1)	69.0, 223.5	134.1 (28.9)	59.0, 242.0
M2	MinCDF	Gray value corresponding to the 5% region cutoff on cumulative density function; higher values correspond to denser region	**-0.13**	**0.04**	**0.23**	**0.0005**	98.0 (23.6)	35.0, 162.0	78.1 (27.3)	15.0, 210.0
M3	Balance	Ratio of (95% CDF-AVE) to (AVE-5% CDF); related to skewness; values less than one correspond to having an ROI that is skewed toward relatively denser values	**-0.32**	**<0.0001**	-0.04	0.49	1.07 (0.42)	0.45, 3.31	1.11 (0.40)	0.38, 2.33
**Texture-based features:**										
T1	Energy	Measure of image homogeneity; higher values correspond to being more homogeneous	**-0.30**	**<0.0001**	**0.19**	**0.003**	0.004 (0.011)	0.0, 0.109	0.003 (0.004)	0.0, 0.028
T2	MaxF (COOC)	Largest number of a gray value pair in the co-occurrence matrix; measure of image homogeneity; higher values correspond to being more homogeneous	**-0.24**	**0.0002**	**0.15**	**0.02**	0.012 (0.024)	0.001, 0.239	0.010 (0.017)	0.001, 0.145

CDF, cumulative density function; COOC, co-occurrence; ROI, region-of-interest. *Spearman’s rank correlation coefficient. ^1^All features were selected using the training dataset. The Balance feature was only selected in sensitivity analyses where the training dataset was truncated at the upper age-limit of mutation carriers. P-values <0.05 are shown in bold font.

The selected gray-level magnitude-based features (AVE, MinCDF, and Balance) were strongly correlated with each other; however, of the three gray-level magnitude-based features, only MinCDF was statistically significantly and positively correlated with the two selected texture-based features (Additional file 1: Table S2). The selected texture-based features, Energy and MaxF (COOC), were strongly and positively correlated with one another (*r* = 0.90, *P* <0.0001) (Additional file 1: Table S2). There were no statistically significant mean differences in the selected computer-extracted feature measures between the training and testing data sets (*P* -value range from Wilcoxon rank sum test = 0.17 to 0.45; data not shown). Likewise, the descriptive characteristics of and correlations between the selected computer extracted features in the testing dataset were consistent with those observed for the training and testing datasets combined (data not shown).

### Relationships between computer-extracted mammographic features and BRCA1/2 mutation status: original training and testing datasets

Table 3 shows the results for the ability of the BANN-trained classifier, developed using the selected feature subset, to distinguish between *BRCA1/2* mutation carriers and non-carriers in the independent testing dataset. The AUC (standard error, SE) for the BANN-trained classifier of 0.68 (0.07) was an improvement over the AUC (SE) for PMD alone (0.59 (0.07)); however, the two AUC statistics were not significantly different from one another (*P* = 0.52), likely due to the small sample size. One SD increase in the probability score from the BANN-trained classifier, developed using the features selected in the original training dataset, was associated in the testing data with about a two-fold increase in the odds of predicting *BRCA1/2* mutation status in both unadjusted (odds ratio (OR) = 2.00, 95% CI: 1.59, 2.51, *P* = 0.02) and age-adjusted (OR = 1.93, 95% CI: 1.53, 2.42, *P* = 0.03) models. Additional adjustment for PMD did not alter the observed age-adjusted OR. The findings were nearly identical when the BANN-trained classifier, modified to include PMD (that is, Features + PMD in Table 3), was used, and when adjusting for baseline characteristics that differed by mutation status (that is, parity, age at first birth, oral contraceptive use, and surgical menopause) (data not shown).

**Table 3:** **Ability of trained classifier to distinguish between** ***BRCA1/2*** **mutation carriers and non-carriers in testing dataset**

Training dataset*	Testing dataset		Testing dataset results										
Description	Number of non-carriers	Number of carriers	Odds ratio	95% CI	***P***-value	Odds ratio	95% CI	***P***-value	OR	95% CI	***P***-value	AUC	SE
			***Unadjusted***			***Adjusted for age***			***Adjusted for age and PMD***				
Percent mammographic density (PMD) alone	30	30	1.02^2^	(0.99, 1.06)	0.21	1.00^2^	(0.96, 1.04)	0.96	N/A			0.59	0.07
Features alone^1^	30	30	**2.00** ^**3**^	**(1.59, 2.51)**	**0.02**	**1.93** ^**3**^	**(1.53, 2.42)**	**0.03**	**1.93** ^**3**^	**(1.54, 2.43)**	**0.03**	0.68	0.07
Features^1^ + PMD	30	30	**2.10** ^**3**^	**(1.67, 2.65)**	**0.01**	**2.03** ^**3**^	**(1.62, 2.56)**	**0.03**	N/A			0.72	0.07

*Training dataset includes 70 non-carriers and 107 *BRCA1/2* mutation carriers. ^1^Four features were selected by the trained classifier: MinCDF, Energy, AVE, and MaxF (COOC); percent mammographic density was not selected by the trained classifier but was forced into the models where noted. ^2^Odds ratios, per unit increase in percent mammographic density. ^3^Odds ratios, per one SD increase in probability score from trained classifier; SD from both models = 0.342. AUC, area under the curve; N/A, not applicable; PMD, percent mammographic density; SE, standard error. P-values <0.05 are shown in bold font.

### Relationships between computer-extracted mammographic features and BRCA1/2 mutation status: sensitivity analyses utilizing an age-matched testing dataset

By virtue of the Breast Imaging Study eligibility criteria, the *BRCA1/2* mutation carriers were on average approximately 10 years younger than the non-carriers. We therefore conducted a series of age-matched sensitivity analyses. First, the testing dataset was restricted to pairs of *BRCA1/2* mutation carriers and non-carriers matched on age within ±3 years (Additional file 1: Table S3). The mean paired differences in the probability scores from the trained classifiers developed using selected features alone and the features plus PMD were statistically significantly greater than zero (*P* = 0.02 and *P* = 0.02, respectively). Using the same age-matched testing dataset, the corresponding AUC (SE) values for the BANN-trained classifier without and with PMD were 0.71 (0.09) and 0.72 (0.08), respectively. When matching on age within ±1 year, the findings were similar, although the mean paired difference in the probability score was no longer statistically significant (*P* = 0.06 for features alone and *P* = 0.08 for features + PMD). Computing AUCs within age strata yielded comparable results (data not shown).

We performed additional sensitivity analyses by removing women above age 55 years (the upper limit of age among the Breast Imaging Study participants) from both the training and testing datasets. This resulted in 96 women in the training data (48 mutation carriers and 48 non-carriers) and 38 women in the testing data (19 carriers and 19 non-carriers) matched on age within ±3 years. The mean paired difference in the probability score from the BANN-trained classifier, developed using the newly-selected feature subset (MinCDF, MaxF (COOC), Balance), was of borderline statistical significance (*P* = 0.055). Forcing PMD into the BANN-trained classifier did not substantially alter our ability to distinguish between *BRCA1/2* mutation carriers and non-carriers (*P* = 0.06). The AUC (SE) for the BANN-trained classifier to distinguish between *BRCA1/2* mutation carriers and non-carriers was 0.72 (0.09) for features alone and 0.71 (0.09) for the features plus PMD. The results from these sensitivity analyses are consistent with those from our primary analyses based on the original testing dataset.

In contrast to the differences we observed in the BANN-trained classifier between *BRCA1/2* mutation carriers and non-carriers, we did not observe any statistically significant mean paired differences in PMD between the test-set pairs age-matched within ±3 or ±1 years or when using the age-restricted dataset (Additional file 1: Table S3, *P* = 0.83, *P* = 1.00, and *P* = 0.83, respectively).

## Discussion

We investigated relationships between computer-extracted mammographic texture features and *BRCA1/2* mutation status among women without breast cancer, and identified novel mammographic texture features (AVE, MinCDF, Energy, MaxF (COOC)) that appear to distinguish *BRCA1/2* mutation carriers from non-carriers. We had previously observed no difference in percent density obtained from the entire mammogram by *BRCA1/2* mutation status in this same population, motivating our search for new informative parenchymal characteristics based on radiographic texture analysis within a retro-areolar ROI. These associations changed minimally when we included PMD in models with the four selected texture features. Thus, the associations we have identified between specific RTA features and mutation status are independent from any possible modifying effect of mammographic density, which in both our prior work and that of others appears no different in mutation carriers than that observed in the general population [12],[14]-[18]. The strength of the RTA feature associations was attenuated when mutation carriers were age-matched to non-carriers, likely due to reduced sample size. Our study adds to the existing RTA literature [19],[20] by analyzing the largest number of mutation carriers yet studied in this manner, and our findings indicate that computer-extracted mammographic features provide some additional information for identifying women likely to carry *BRCA1*/*2* mutations. The RTA classifier we have identified could prove a useful adjunct to mammographic interpretation both in women from families with many affected relatives in whom no genetic susceptibility has yet been identified and in families known to have mutations in these genes. However, because the positive predictive value of such a test would be low in the general population, owing to the rarity of these mutations, the strength of the association we found is not high enough for screening a general population to identify candidates for mutation testing.

The texture-based features Energy and MaxF (COOC) - which describe the spatial distribution pattern for tissue homogeneity - and AVE and MinCDF - which provide gray-level magnitude information on tissue denseness - were the strongest RTA predictors of mutation status within a given ROI. The RTA texture-based features selected in this study characterize similar parenchymal attributes found in previous studies on digitized screen/film mammograms [19],[27],[28],[30], such that *BRCA1/2* mutation carriers tend to have retro-areolar parenchymal patterns that are coarse in texture. It is important to note that a given parenchymal attribute may be described by multiple computer-extracted features. For example, image homogeneity can be measured by Energy and the largest number of a gray-value pair in the co-occurrence matrix (MaxF (COOC)), as selected in this study, or by the first moment of the power spectrum (FMP) or Coarseness, which Huo *et al*. and Li *et al*. previously found to be associated with *BRCA1/2* mutation status [19],[27]. In addition, our findings are consistent with two case-control studies reporting that mammograms of coarse texture are associated with increased breast cancer risk [23],[24]. In these studies, however, simultaneous inclusion of the texture features in a model with PMD did not improve breast cancer risk prediction [23],[24].

Although we found that the selected texture features significantly improved our ability to distinguish between mutation carriers and non-carriers when compared with PMD alone, ours was a cross-sectional study evaluating features associated with *BRCA1/2* mutation status rather than subsequent risk of developing breast cancer. Prior studies have questioned the importance of mammographic density for breast cancer risk prediction among *BRCA1/2* mutation carriers [11]; further research is warranted to investigate the predictive value of computer-extracted texture features among this high-risk patient population. We currently have no information on the association between the RTA classifier and the risk of breast cancer *per se* among *BRCA* mutation carriers. While it may seem logical to assume that women with the *BRCA*-related RTA mammographic texture pattern will actually be at increased risk of breast cancer, that fact has not yet been established. Further clinical development of the RTA classifier will require proof of this hypothesized association; we strongly recommend that a new study with that question as its primary study endpoint be undertaken.

Mutation carriers tended to have lower values for the RTA gray-level magnitude-based features selected in this study, suggesting that their breasts were less dense in the retro-areolar region as compared with the non-carriers. This finding is inconsistent with prior studies suggesting that mutation carriers have gray-level magnitude-based features that are low in contrast [19],[30]. It is possible that differences in film digitizers and/or digital mammographic image acquisition systems between studies could influence RTA, particularly for the gray-level magnitude-based features which have been previously shown to be sensitive to the effects of variable gain [48]. Consistent with the idea that texture-based features are more robust than gray-level magnitude-based features across systems of varying gain [48], a prior study, which utilized full-field digital mammograms (FFDM) to identify high-risk features, resulted in selection of only spatial distribution texture-based features [49]. Hence, the gray-level magnitude-based features that were related to mutation status in our study population may not be generalizable to FFDM. This is not surprising as image processing of FFDM permits the degree of contrast in the image to be manipulated, such that contrast may be increased in the dense areas of the breast in order to maximize mammographic sensitivity [50]. As clinical practice is rapidly shifting toward digital breast imaging, this work should set the stage for applying the strategies described herein to newer images from mutation carriers as they become available.

Our research method was also limited by the need for manual placement of retro-areolar ROIs; however, manual ROI reselection for a randomly selected subset of participants was found to be highly reliable, both in this study and as reported previously [20]. Automation of ROI placement could be applied in future work. Our study had several strengths, including the largest number of mutation carriers and non-carriers yet studied in this manner, assessment of digitized images that was completely masked to mutation status and evaluation of the proposed classifier in independent test data. Although the discriminatory accuracy of the RTA classifier was modest (AUC = 0.68), and for a diagnostic test we would like to have a higher value, the AUC does compare favorably with AUC statistics reported in most breast cancer risk models [51]. Further, we performed extensive sensitivity analyses, and our findings persisted in the presence of multiple potential confounding factors, including age and PMD. Although statistical power was limited for the age-matched sensitivity analyses, these analyses provided an important confirmatory way to control for age and results were consistent in their suggestion of a relation between computer-extracted mammographic texture pattern features and mutation status. Thus, our findings warrant validation in larger independent clinical studies.

The biology of mammographic density is poorly understood [52],[53], and the biologic correlates of texture-based features are even less well-characterized. Nevertheless, evidence from animal models and human breast tissues suggests underlying biological differences in the molecular histology and pathology of the breast by *BRCA1/2* mutation status [54]-[57]. While it is possible that our results may be related to true anatomical differences between carriers and non-carriers as reflected in their parenchymal patterns, other biologic factors, such as biochemical differences, also need to be explored.

## Conclusions

Several noteworthy clinical implications flow from our results. First, we confirm an important observation, previously made by Huo *et al*. and Li *et al*. [19],[20] but not widely appreciated in the clinical community: the digitized mammographic image contains computer-extractable information not captured during routine radiologic interpretation which may permit improved, real-time risk stratification among women undergoing screening mammography. Nonetheless, it is early days for the tools used in this analysis; further development of these techniques might identify additional, more strongly-correlated features. In the current instance, our computer model was significantly correlated with the presence of deleterious mutations in *BRCA1/2*, conferring a two-fold increase in the likelihood of being a mutation carrier, per one SD increase in the probability score*.* If the interpreting radiologist were to be made aware of this information while reading clinical mammographic images, it could alter image interpretation by increasing the prior probability of disease in subjects with the *BRCA*-related pattern. The model’s ability to distinguish between *BRCA1/2* mutation carriers and non-carriers might, in the context of a positive family history of breast and/or ovarian cancer, serve as an indicator to consider formal genetic risk assessment in persons who have not been previously tested. Integration of breast imaging data with family history and breast tumor markers could be formally assessed by estimating the added value of our image-based probability score to existing statistical models that are used to predict *BRCA1/2* mutations [58]. Although mathematical and statistical concepts involved in generating the RTA classifier are complex, a great deal of work has already been done relative to the details of this methodology. Should the RTA classifier be validated clinically, this algorithm is amenable to a user-friendly implementation. The current data do not support these clinical applications at the present time, but they provide a solid basis for extending this novel research into larger, more rigorously-designed studies utilizing digital imaging modalities. Our findings also serve as a reminder of the importance of keeping an open mind relative to novel applications of old technologies. This value-added strategy may improve the cost-benefit ratios of tried, true and readily available clinical tests, without the development costs associated with an entirely new technology.

## Additional files

## Electronic supplementary material

Additional file 1: Table S1.: Intraclass correlation coefficients (ICC) for masked reliability assessment of Computer-extracted features (n = 91 pairs). **Table S2.** Correlations between selected computer-extracted features (n = 237 women). **Table S3.** Sensitivity analyses of the ability of the trained classifier to distinguish between *BRCA1/2* mutation carriers and non-carriers in age-matched datasets. **Figure S1.** Histogram of the number of times that each feature was selected in the 177 leave-one-case-out stepwise feature selection using linear discriminant analysis of the training dataset. (DOC 304 KB)

Additional file 2: **Appendix 1.** Stepwise Feature Selection using Linear Discriminant Analysis. **Appendix 2.** Bayesian Artificial Neural Networks. (DOC 200 KB)

Below are the links to the authors’ original submitted files for images.

Authors’ original file for figure 1

Authors’ original file for figure 2

Authors’ original file for figure 3

## Abbreviations

AUC: area under curve
BANN: Bayesian artificial neural network
CDF: cumulative distribution function
COOC: co-occurrence
FFDM: full-field digital mammogram
ICC: intraclass correlation coefficient
IRB: Institutional Review Board
NCI: National Cancer Institute
NIH: National Institutes of Health
NNMC: National Naval Medical Center
OR: odds ratio
PAT: Pedigree Assessment Tool
PMD: percent mammographic density
ROC: receiver operating characteristic
ROI: region-of-interest
RTA: radiographic texture analysis

## References

[1] Boyd NF, Lockwood GA, Byng JW, Tritchler DL, Yaffe MJ (1998). Mammographic densities and breast cancer risk. Cancer Epidemiol Biomarkers Prev.

[2] Haars G, van Noord PA, van Gils CH, Peeters PH, Grobbee DE (2004). Heritable aspects of dysplastic breast glandular tissue (DY). Breast Cancer Res Treat.

[3] Kaprio J, Alanko A, Kivisaari L, Standertskjold-Nordenstam CG (1987). Mammographic patterns in twin pairs discordant for breast cancer. Br J Radiol.

[4] Kataoka M, Antoniou A, Warren R, Leyland J, Brown J, Audley T, Easton D (2009). Genetic models for the familial aggregation of mammographic breast density. Cancer Epidemiol Biomarkers Prev.

[5] Pankow JS, Vachon CM, Kuni CC, King RA, Arnett DK, Grabrick DM, Rich SS, Anderson VE, Sellers TA (1997). Genetic analysis of mammographic breast density in adult women: evidence of a gene effect. J Natl Cancer Inst.

[6] Vachon CM, King RA, Atwood LD, Kuni CC, Sellers TA (1999). Preliminary sibpair linkage analysis of percent mammographic density. J Natl Cancer Inst.

[7] Vachon CM, Sellers TA, Carlson EE, Cunningham JM, Hilker CA, Smalley RL, Schaid DJ, Kelemen LE, Couch FJ, Pankratz VS (2007). Strong evidence of a genetic determinant for mammographic density, a major risk factor for breast cancer. Cancer Res.

[8] Wolfe JN, Albert S, Belle S, Salane M (1980). Familial influences on breast parenchymal patterns. Cancer.

[9] Boyd N, Dite G, Stone J, Gunasekara A, English D, McCredie M (2002). Heritability of mammographic density, a risk factor for breast cancer. N Engl J Med.

[10] Ursin G, Lillie EO, Lee E, Cockburn M, Schork NJ, Cozen W, Parisky YR, Hamilton AS, Astrahan MA, Mack T (2009). The relative importance of genetics and environment on mammographic density. Cancer Epidemiol Biomarkers Prev.

[11] Passaperuma K, Warner E, Hill KA, Gunasekara A, Yaffe MJ (2010). Is mammographic breast density a breast cancer risk factor in women with BRCA mutations?. J Clin Oncol.

[12] Mitchell G, Antoniou AC, Warren R, Peock S, Brown J, Davies R, Mattison J, Cook M, Warsi I, Evans DG, Eccles D, Douglas F, Paterson J, Hodgson S, Izatt L, Cole T, Burgess L, Eeles R, Easton DF (2006). Mammographic density and breast cancer risk in *BRCA1* and *BRCA2*mutation carriers. Cancer Res.

[13] Clark AS, Domchek SM (2011). Clinical management of hereditary breast cancer syndromes. J Mammary Gland Biol Neoplasia.

[14] Gierach GL, Loud JT, Chow CK, Prindiville SA, Eng-Wong J, Soballe PW, Giambartolomei C, Mai PL, Galbo CE, Nichols K, Calzone KA, Vachon C, Gail MH, Greene MH (2010). Mammographic density does not differ between unaffected *BRCA1/2*mutation carriers and women at low-to-average risk of breast cancer. Breast Cancer Res Treat.

[15] Helvie MA, Roubidoux MA, Weber BL, Merajver SD (1997). Mammography of breast carcinoma in women who have mutations of the breast cancer gene *BRCA1*: initial experience. Am J Roentgenol.

[16] Tilanus-Linthorst M, Verhoog L, Obdeijn IM, Bartels K, Menke-Pluymers M, Eggermont A, Klijn J, Meijers-Heijboer H, van der Kwast T, Brekelmans C (2002). A *BRCA1/2*mutation, high breast density and prominent pushing margins of a tumor independently contribute to a frequent false-negative mammography. Int J Cancer.

[17] Hamilton LJ, Evans AJ, Wilson AR, Scott N, Cornford EJ, Pinder SE, Khan HN, Macmillan RD (2004). Breast imaging findings in women with *BRCA1*- and *BRCA2*-associated breast carcinoma. Clin Radiol.

[18] Kaas R, Kroger R, Peterse JL, Hart AA, Muller SH (2006). The correlation of mammographic-and histologic patterns of breast cancers in *BRCA1*gene mutation carriers, compared to age-matched sporadic controls. Eur Radiol.

[19] Huo Z, Giger ML, Olopade OI, Wolverton DE, Weber BL, Metz CE, Zhong W, Cummings SA (2002). Computerized analysis of digitized mammograms of *BRCA1* and *BRCA2*gene mutation carriers. Radiology.

[20] Li H, Giger ML, Huo Z, Olopade OI, Lan L, Weber BL, Bonta I (2004). Computerized analysis of mammographic parenchymal patterns for assessing breast cancer risk: effect of ROI size and location. Med Phys.

[21] Yaffe M (2008). Mammographic density: measurement of mammographic density. Breast Cancer Res.

[22] Byng JW, Yaffe MJ, Lockwood GA, Little LE, Tritchler DL, Boyd NF (1997). Automated analysis of mammographic densities and breast carcinoma risk. Cancer.

[23] Haberle L, Wagner F, Fasching PA, Jud SM, Heusinger K, Loehberg CR, Hein A, Bayer CM, Hack CC, Lux MP, Binder K, Elter M, Munzenmayer C, Schulz-Wendtland R, Meier-Meitinger M, Adamietz BR, Uder M, Beckmann MW, Wittenberg T (2012). Characterizing mammographic images by using generic texture features. Breast Cancer Res.

[24] Manduca A, Carston MJ, Heine JJ, Scott CG, Pankratz VS, Brandt KR, Sellers TA, Vachon CM, Cerhan JR (2009). Texture features from mammographic images and risk of breast cancer. Cancer Epidemiol Biomarkers Prev.

[25] Nielsen M, Karemore G, Loog M, Raundahl J, Karssemeijer N, Otten JDM, Karsdal MA, Vachon CM, Christiansen C (2011). A novel and automatic mammographic texture resemblance marker is an independent risk factor for breast cancer. Cancer Epidemiol.

[26] Wei J, Chan H-P, Wu Y-T, Zhou C, Helvie MA, Tsodikov A, Hadjiiski LM, Sahiner B (2011). Association of computerized mammographic parenchymal pattern measure with breast cancer risk: a pilot case-control study. Radiology.

[27] Li H, Giger ML, Olopade OI, Chinander MR (2008). Power spectral analysis of mammographic parenchymal patterns for breast cancer risk assessment. J Digit Imaging.

[28] Li H, Giger ML, Olopade OI, Lan L (2007). Fractal analysis of mammographic parenchymal patterns in breast cancer risk assessment. Acad Radiol.

[29] Li H, Giger ML, Olopade OI, Margolis A, Lan L, Chinander MR (2005). Computerized texture analysis of mammographic parenchymal patterns of digitized mammograms. Acad Radiol.

[30] Huo Z, Giger ML, Wolverton DE, Zhong W, Cumming S, Olopade OI (2000). Computerized analysis of mammographic parenchymal patterns for breast cancer risk assessment: feature selection. Med Phys.

[31] Yaffe MJ, Boyd NF, Byng JW, Jong RA, Fishell E, Lockwood GA, Little LE, Tritchler DL (1998). Breast cancer risk and measured mammographic density. Eur J Cancer Prev.

[32] Torres-Mejia G, De Stavola B, Allen DS, Perez-Gavilan JJ, Ferreira JM, Fentiman IS, Dos Santos Silva I (2005). Mammographic features and subsequent risk of breast cancer: a comparison of qualitative and quantitative evaluations in the Guernsey prospective studies. Cancer Epidemiol Biomarkers Prev.

[33] Loud JT, Beckjord EB, Nichols K, Peters J, Giusti R, Greene MH (2009). Tolerability of breast ductal lavage in women from families at high genetic risk of breast cancer. BMC Womens Health.

[34] Loud JT, Thiebaut AC, Abati AD, Filie AC, Nichols K, Danforth D, Giusti R, Prindiville SA, Greene MH (2009). Ductal lavage in women from *BRCA1/2*families: is there a future for ductal lavage in women at increased genetic risk of breast cancer?. Cancer Epidemiol Biomarkers Prev.

[35] Gail MH, Brinton LA, Byar DP, Corle DK, Green SB, Schairer C, Mulvihill JJ (1989). Projecting individualized probabilities of developing breast cancer for white females who are being examined annually. J Natl Cancer Inst.

[36] Hoskins KF, Zwaagstra A, Ranz M (2006). Validation of a tool for identifying women at high risk for hereditary breast cancer in population-based screening. Cancer.

[37] Nelson HD, Fu R, Goddard K, Mitchell JP, Okinaka-Hu L, Pappas M, Zakher B (2013). Risk Assessment, Genetic Counseling, and Genetic Testing for BRCA-Related Cancer: Systematic Review to Update the U.S. Preventive Services Task Force Recommendation. Evidence Synthesis No. 101. AHRQ Publication No. 12-05164-EF-1.

[38] Teller P, Hoskins KF, Zwaagstra A, Stanislaw C, Iyengar R, Green VL, Gabram SG (2010). Validation of the pedigree assessment tool (PAT) in families with *BRCA1* and *BRCA2*mutations. Ann Surg Oncol.

[39] Sonka M, Hlavac V, Boyle R (1999). Image Processing, Analysis, and Machine Vision.

[40] Chen W, Giger ML, Li H, Bick U, Newstead GM (2007). Volumetric texture analysis of breast lesions on contrast-enhanced magnetic resonance images. Magn Reson Med.

[41] Haralick RM, Shanmugan K, Dinstein I (1973). Textural features for image classification. IEEE Trans Syst Man Cybern.

[42] Huberty CJ (1994). Applied Discriminant Analysis.

[43] Lachenbruch PA (1975). Discriminant Analysis.

[44] Metz CE (1989). Some practical issues of experimental design and data analysis in radiological ROC studies. Invest Radiol.

[45] Metz CE, Herman BA, Shen JH (1998). Maximum likelihood estimation of receiver operating characteristic (ROC) curves from continuously-distributed data. Stat Med.

[46] ROCKIT, version 1.1b. [http://metz-roc.uchicago.edu/MetzROC/software]

[47] Metz CE, Herman BA, Roe CA (1998). Statistical comparison of two ROC-curve estimates obtained from partially-paired datasets. Med Decis Making.

[48] Li H, Giger ML, Lan L, Yuan Y, Bhooshan N, Olopade O (2010). Effect of variable gain on computerized texture analysis on digitized mammograms. Proc SPIE Med Imag Conf.

[49] Li H, Giger ML, Lan L, Bancroft Brown J, MacMahon A, Mussman M, Olopade OI, Sennett C (2012). Computerized analysis of mammographic parenchymal patterns on a large clinical dataset of full-field digital mammograms: robustness study with two high-risk datasets. J Digit Imaging.

[50] Pisano ED, Gatsonis C, Hendrick E, Yaffe M, Baum JK, Acharyya S, Conant EF, Fajardo LL, Bassett L, D’Orsi C, Jong R, Rebner M (2005). Digital Mammographic Imaging Screening Trial Investigators Group: **Diagnostic performance of digital versus film mammography for breast-cancer screening**. N Engl J Med.

[51] Cummings SR, Tice JA, Bauer S, Browner WS, Cuzick J, Ziv E, Vogel V, Shepherd J, Vachon C, Smith-Bindman R, Kerlikowske K (2009). Prevention of breast cancer in postmenopausal women: approaches to estimating and reducing risk. J Natl Cancer Inst.

[52] Martin LJ, Boyd NF (2008). Mammographic density - Potential mechanisms of breast cancer risk associated with mammographic density: hypotheses based on epidemiological evidence. Breast Cancer Res.

[53] Sun X, Gierach GL, Sandhu R, Williams T, Midkiff BR, Lissowska J, Wesolowska E, Boyd NF, Johnson NB, Figueroa JD, Sherman ME, Troester MA (2013). Relationship of mammographic density and gene expression: analysis of normal breast tissue surrounding breast cancer. Clin Cancer Res.

[54] Lim E, Vaillant F, Wu D, Forrest NC, Pal B, Hart AH, Asselin-Labat ML, Gyorki DE, Ward T, Partanen A, Feleppa F, Huschtscha LI, Thorne HJ, kConFab, Fox SB, Yan M, French JD, Brown MA, Smyth GK, Visvader JE, Lindeman GJ (2009). Aberrant luminal progenitors as the candidate target population for basal tumor development in *BRCA1*mutation carriers. Nat Med.

[55] Mavaddat N, Barrowdale D, Andrulis IL, Domchek SM, Eccles D, Nevanlinna H, Ramus SJ, Spurdle A, Robson M, Sherman M, Mulligan AM, Couch FJ, Engel C, McGuffog L, Healey S, Sinilnikova OM, Southey MC, Terry MB, Goldgar D, O'Malley F, John EM, Janavicius R, Tihomirova L, Hansen TV, Nielsen FC, Osorio A, Stavropoulou A, Benitez J, Manoukian S, Peissel B (2012). Pathology of breast and ovarian cancers among *BRCA1* and *BRCA2* mutation carriers: results from the Consortium of Investigators of Modifiers of *BRCA1/2*(CIMBA). Cancer Epidemiol Biomarkers Prev.

[56] Silver DP, Livingston DM (2012). Mechanisms of **BRCA1**tumor suppression. Cancer Discovery.

[57] Roy R, Chun J, Powell SN (2012). *BRCA1* and *BRCA2*: different roles in a common pathway of genome protection. Nat Rev Cancer.

[58] Antoniou AC, Hardy R, Walker L, Evans DG, Shenton A, Eeles R, Shanley S, Pichert G, Izatt L, Rose S, Douglas F, Eccles D, Morrison PJ, Scott J, Zimmern RL, Easton DF, Pharoah PD (2008). Predicting the likelihood of carrying a *BRCA1* or *BRCA2*mutation: validation of BOADICEA, BRCAPRO, IBIS, Myriad and the Manchester scoring system using data from UK genetics clinics. J Med Genet.

