# Supplementary material for: Relationships between computer-extracted mammographic texture pattern features and BRCA1/2mutation status: a cross-sectional study
Source: Breast Cancer Res. 2014 Aug 23;16:424. doi: 10.1186/s13058-014-0424-8 (PMC4268674; doi:10.1186/s13058-014-0424-8)
Supplement: Supplementary file 1 — Additional file 1: Table S1.: Intraclass correlation coefficients (ICC) for masked reliability assessment of Computer-extracted features (n = 91 pairs). Table S2. Correlations between selected computer-extracted features (n = 237 women). Table S3. Sensitivity analyses of the ability of the trained classifier to distinguish between BRCA1/2 mutation carriers and non-carriers in age-matched datasets. Figure S1. Histogram of the number of times that each feature was selected in the 177 leave-one-case-out stepwise feature selection using linear discriminant analysis of the training dataset. (DOC 304 KB) [file 13058_2014_424_MOESM1_ESM.doc]

| **Supplementary Table 1. Intraclass correlation coefficients (ICC) for masked reliability assessment of computer-extracted features (n=91 pairs)** | | | | |
| --- | --- | --- | --- | --- |
| **Feature Type and Number** | **Feature** | **Definition** | **ICC** | **Reference** |
| **SELECTED FEATURES:** |  |  |  |  |
| **Gray level magnitude-based features:** |  |  |  |  |
| M1 | AVE | Average gray value within ROI; higher values correspond to denser region | 0.986 | [1-3] |
| M2 | MinCDF | Gray value corresponding to the 5% region cutoff on cumulative density function; higher values correspond to denser region | 0.971 | [1-3] |
| M3 | Balance | Ratio of (95%CDF-AVE) to (AVE-5%CDF); Related to skewness; Values less than one correspond to having a ROI that is skewed towards relatively denser values. | 0.959 | [1-4] |
| **Texture-based features:** |  |  |  |  |
| T1 | Energy | Measure of image homogeneity; higher values correspond to being more homogeneous | 0.980 | [5-7] |
| T2 | MaxF (COOC) | Largest number of a gray value pair in the co-occurrence matrix; measure of image homogeneity; higher values correspond to being more homogeneous | 0.987 | [5-7] |
| **OTHER FEATURES:** |  |  |  |  |
| **Gray level magnitude-based features:** |  |  |  |  |
| M4 | MaxCDF | Gray value corresponding to the 95% region cutoff on cumulative density function; higher values correspond to denser region | 0.996 | [1-3] |
| M5 | 70%CDF | Gray value corresponding to the 70% region cutoff on cumulative density function; higher values correspond to denser region | 0.989 | [1-3] |
| M6 | 30%CDF | Gray value corresponding to the 30% region cutoff on cumulative density function; higher values correspond to denser region | 0.982 | [1-3] |
| M7 | Balance2 | Ratio of (70%CDF-AVE) to (AVE-30%CDF); Related to skewness; Values larger than one correspond to denser regions | 0.842 | [1-4] |
| M8 | Skewness | The denseness measure; Negative values correspond to denser region | 0.981 | [1-4] |
| M9 | RMS | Rood mean square variation; quantifies the magnitude of parenchymal patterns | 0.990 | [1-3, 7] (cont’d) |

**Additional file 1**

| **Feature Type and Number** | **Feature** | **Definition** | **ICC** | **Reference** |
| --- | --- | --- | --- | --- |
| **OTHER FEATURES:** |  |  |  |  |
| **Texture-based features:** |  |  |  |  |
| T3 - T8 | D_BC [1-6] | Fractal dimension estimated based on box-counting method; lower values correspond to coarser texture | Range:  0.895-0.995 | [3, 8] |
| T9 | D_M | Fractal dimension estimated based on Minkowski method; lower values correspond to coarser texture | 0.993 | [3, 8] |
| T10 - T17 | Beta [1-8] | Exponent beta from power law spectrum analysis; characterize the frequency content of texture pattern; higher values correspond to coarser texture | Range:  0.960-0.990 | [9] |
| T18 | Contrast (COOC) | Contrast measure calculated from co-occurrence matrix; measure of image local variations | 0.998 | [5-7] |
| T19 | Contrast (NGTDM) | Contrast measure calculated from Neighborhood-Gray-tone-difference matrix; measure of image local variations | 0.989 | [1-4] |
| T20 | Correlation (COOC) | Measure of image linearity; larger values correspond to linear patterns | 0.791 | [5-7] |
| T21 | Entropy (COOC) | Measure of randomness of gray level pairs | 0.996 | [5-7] |
| T22 | ZeroMeasure (COOC) | Zero measures in co-occurrence matrix; measure of image homogeneity | 0.982 | [5-7] |
| T23 | Skewness (COOC) | Measure of the asymmetry of co-occurrence matrix; image homogeneity measure | 0.983 | [5-7] |
| T24 | MeanEdgeGradient | Average of edge gradient; image coarseness measure | 0.997 | [3, 7] |
| T25 | MaxEdgeGradient | Maximum edge gradient; image coarseness measure | 0.905 | [3, 7] |
| T26 | MinEdgeGradient | Minimum edge gradient; image coarseness measure | 0.933 | [3, 7] |
| T27 | StdDevEdgeGradient | Standard deviation of edge gradient; image coarseness measure | 0.996 | [3, 7] |
| T28 | Coarseness (NGTDM) | Measure of image coarseness; higher values correspond to coarser region | 0.953 | [1-4] |
| T29 | FMP | First moment of power spectrum; spatial frequency content of parenchymal patterns | 0.972 | [1-3, 7] |
|  |  |  |  |  |


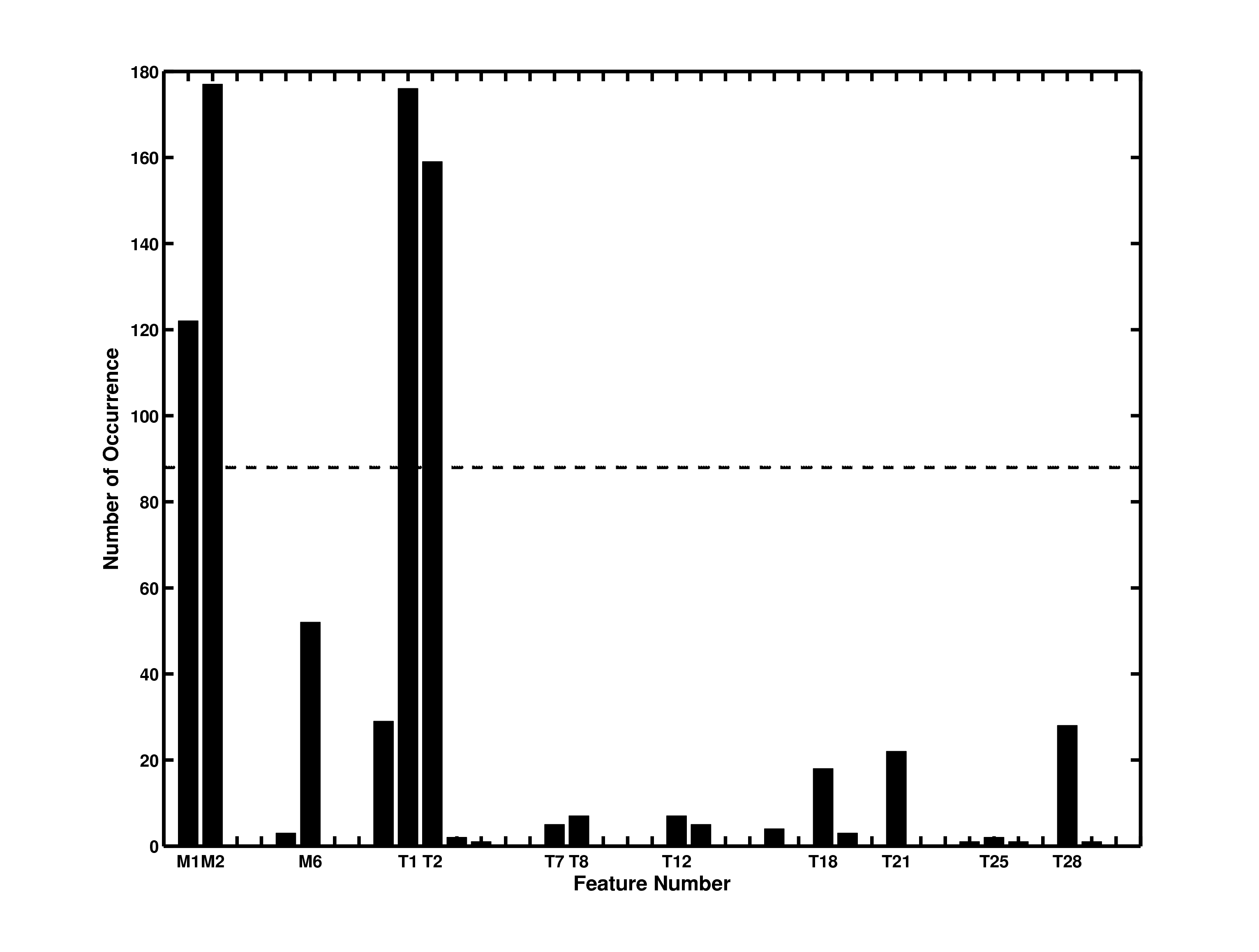
**Supplementary Figure 1**. Histogram of the number of times that each feature was selected in the 177 leave-one-case-out stepwise feature selection using linear discriminant analysis of the training dataset. Note the features M1-M9 correspond to gray level magnitude-based features, and features T1-T29 correspond to texture-based features. Two gray level magnitude-based features (*i.e.,* M1, AVE; M2, MinCDF) and two texture-based features (*i.e.,* T1, Energy; T2, MaxF (COOC)) were selected more than half the time (as indicated by the dashed line in the figure), and were included in subsequent Bayesian Artificial Neural Network models.

**References**

1. Huo Z, Giger ML, Wolverton DE*, et al.* Computerized analysis of mammographic parenchymal patterns for breast cancer risk assessment: feature selection. *Med Phys* 2000;27(1):4-12.

2. Li H, Giger ML, Huo Z*, et al.* Computerized analysis of mammographic parenchymal patterns for assessing breast cancer risk: effect of ROI size and location. *Med Phys* 2004;31(3):549-555.

3. Li H, Giger ML, Olopade OI*, et al.* Computerized Texture Analysis of Mammographic Parenchymal Patterns of Digitized Mammograms. *Academic Radiology* 2005;12(7):863-873.

4. Huo Z, Giger ML, Olopade OI*, et al.* Computerized analysis of digitized mammograms of *BRCA1* and *BRCA2* gene mutation carriers. *Radiology* 2002;225(2):519-526.

5. Chen W, Giger ML, Li H*, et al.* Volumetric texture analysis of breast lesions on contrast-enhanced magnetic resonance images. *Magn Reson Med* 2007;58(3):562-571.

6. Haralick RM, Shanmugan K, Dinstein I. Textural Features for Image Classification. *IEEE Transactions on Systems, Man, and Cybernetics* 1973;6:610-621.

7. Sonka M, Hlavac V, Boyle R. *Image Processing, Analysis, and Machine Vision*. Second Edition ed: PWS Publishing; 1999.

8. Li H, Giger ML, Olopade OI*, et al.* Fractal analysis of mammographic parenchymal patterns in breast cancer risk assessment. *Acad Radiol* 2007;14(5):513-521.

9. Li H, Giger ML, Olopade OI*, et al.* Power spectral analysis of mammographic parenchymal patterns for breast cancer risk assessment. *J Digit Imaging* 2008;21(2):145-152.
