# Supplementary material for: Relationships between computer-extracted mammographic texture pattern features and BRCA1/2mutation status: a cross-sectional study
Source: Breast Cancer Res. 2014 Aug 23;16:424. doi: 10.1186/s13058-014-0424-8 (PMC4268674; doi:10.1186/s13058-014-0424-8)
Supplement: Supplementary file 2 — Additional file 2: Appendix 1. Stepwise Feature Selection using Linear Discriminant Analysis. Appendix 2. Bayesian Artificial Neural Networks. (DOC 200 KB) [file 13058_2014_424_MOESM2_ESM.doc]

**Additional file 2: Relationships between Computer-extracted Mammographic Texture Pattern Features and *BRCA1/2* Mutation Status**

**Appendix 1: Stepwise Feature Selection using Linear Discriminant Analysis**

Feature selection is a key step in the development of computerized quantitative imaging analysis scheme. Due to the “curse of dimensionality,” it is often necessary to select a subset of features as input to a classifier to determine, for example, whether or not a subject is a *BRCA1/2* gene-mutation carrier.

The most commonly used feature selection method is the stepwise feature selection using linear discriminant analysis. Features are iteratively added into or removed from the group of selected features based on a feature selection criterion, the Wilks’ lambda . Wilks’ lambda was defined as the ratio of the spread within each class to the spread within the entire dataset. In each iteration step, linear discriminant analysis is used to calculate the discriminant scores, which are then used to compute the Wilks’ lambda.

The Wilks’ lambda is defined using the following equation (E):

(E1)

where $g_{i}$ and $l_{i}$ are the discriminant scores for the *BRCA1/2* mutation carriers and non-carriers, respectively, and $\bar{g}$, $\bar{l}$and $a$ are the mean discriminant scores for the mutation carriers, the non-carriers women, and all subjects, respectively. The number of mutation carriers and non-carriers are$N_{g}$ and $N_{l}$, respectively.

In general, if there are a total of $P$features, then in the first step of stepwise feature selection the performance of each of $P$ features is evaluated using Wilks’ lambda, and the feature with the best performance is selected. In the subsequent steps, assuming that $m$is the number of features that have at one time been added to the selected feature subset vector,$V$, we determine $P-m$linear discriminants by adding each of the remaining $P-m$ to $V$. The feature whose addition most improves the performance of the linear classifier is added to $V$ if its contribution to the performance is statistically significant using *F*-statistics. The feature that contributes least to the performance of linear classifier is removed from $V$ if its contribution is not statistically significant. This process is repeated until no more features are added or removed.

Leave-one-case-out (round-robin) stepwise feature selection using linear discriminant analysis was performed in our analysis. For leave-one-case-out feature selection, a single subject $i$is removed, and then stepwise feature selection is performed on $N-1$subjects, and the resulting selected feature subset is recorded. This procedure is repeated $N$times for all subjects, and the frequency of each selected feature is tabulated (**Supplementary Figure 1**). A feature was included in the classification model if it was selected in at least half of the N leave-one-case-out analyses.

**Appendix 2: Bayesian Artificial Neural Networks**

The following is an overview of the theory of Bayesian artificial neural networks following the work of MacKay , Neal , Bishop and Nabney .

**Artificial Neural Network (ANN)**

The most commonly used artificial neutral network (ANN) in classification problems is the multi-layer perceptron (MLP). We used a two-layer feed-forward MLP (three-tier architecture) for the classification task.


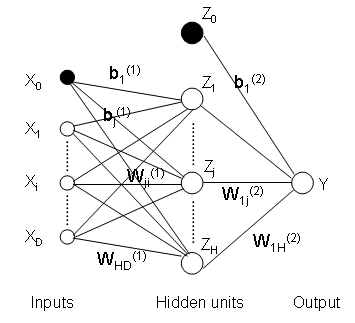


**Schematic diagram of a two-layer artificial neutral network (ANN) with D inputs, H hidden units, and 1 output.**

In the 2-layer feed-forward MLP we used for our analysis shown in the schematic diagram above, the sum of the weighted linear combination of inputs and a bias is transformed by the non-linear activation function, hyperbolic tangent, $\tanh,$of the hidden layer which yields the following equation (E)

$$Z_{j}=\tanh(\sum_{i=1}^{D} w_{ji}^{(1)}x_{i}+b_{j}^{(1)})\text{ , }j=1,...,H\text{ (E1)}$$

where $w_{ji}^{(1)}$ represents a weight in the first layer connecting input *i* to hidden unit *j*, and $b_{j}^{(1)}$ represents the bias associated with the hidden unit *j*. The sum of the weighted linear combination of the hidden layer outputs and a bias is then transformed by another non-linear activation function, which is usually the logistic sigmoidal function in classification neural networks, to yield the output *Y*

$$Y=\frac{1}{1+\exp\{-(\sum_{J=1}^{H} w_{1j}^{(2)}z_{j}+b_{1}^{(2)})\}}\text{ (E2) }$$

where $w_{1j}^{(2)}$represents a weight in the second layer connecting hidden unit *j* to the output, and $b_{1}^{(2)}$ represents the bias associated with the output. For convenience, the four groups of parameters in an MLP were defined as the following:

1) First layer weights:

$$W_{1}=\{w_{ji}^{(1)}\left|i=1,2,...D;j=1,2,...,H\}\right.\text{ (E3)}$$

2) First layer bias:

$$B_{1}=\{b_{j}^{(1)}\left|j=1,2,...,H\}\text{ (E4)}\right.$$

3) Second layer weights:

$$W_{2}=\{w_{1j}^{(2)}\left|j=1,2,...,H\}\text{ (E5)}\right.$$

4) Second layer bias:

$$B_{2}=\{b_{1}^{(2)}\}\text{ (E6)}$$

The entire weight vector $\vec{w}$ in an MLP will be all four-group weight’s union, *i.e.,* $\vec{w}=W_{1}\cup B_{1}\cup W_{2}\cup B_{2}$, and the total number of weights is:

$$nWeights=(nInputs+1)*nHidden+(nHidden+1)*nOutputs\text{ (E7)}$$

In our two-class ($\{C_{g},C_{l}\}$) classification problem, the use of a logistic sigmoidal activation function allows for an interpretation of the output *Y* as the posterior probability of an input belonging to the gene-mutation class$C_{g}$, denoted as$p(C_{g}|\mathop{\vec{x}}\limits)$,

By defining the prevalence parameter$k$and the likelihood ratio$LR(\mathop{\vec{x}}\limits)$:

$$k\equiv\frac{p(C_{g})}{p(C_{l})}\text{ (E9)}$$

The posterior probability can be rewritten as:

It is clear that$p(C_{g}|\mathop{\vec{x}}\limits)$, the Bayes optimal discriminant function is a monotonic transformation of the likelihood ratio. It is well understood that the likelihood ratio or any monotonic transformation of the likelihood ratio is the optimal classification decision variable, or the ideal observer. Therefore, an ANN is theoretically able to represent an ideal observer . Pragmatically, one needs to estimate $\vec{w}$ using a dataset of finite size and can only approximate the optimal discriminant function as. The task of training an ANN is to minimize the difference between the ANN outputand the true Bayes optimal discriminant function$p(C_{g}|\vec{x})$ .

**Bayesian Artificial Neutral Network (BANN)**

For a given network architecture, training of an ANN involves using a training dataset $D\equiv\{X,T\}$ to determine a value of , where $X\equiv\{\vec{x}_{i}\}_{i=1}^{N}$ is the set of training feature vectors, $T\equiv\{t_{i}\}_{i=1}^{N}$is the set of known truth ($C_{g}$or $C_{l}$) for each training feature vector and $N$is the total number of samples in the training dataset. Traditional error-back-propagation methods, which yield a maximum likelihood estimation of $\vec{w}$, the sample Bayes optimal discriminant function, often have the problem of “overfitting” − a phenomenon in which the trained neural network fits the training data well but has little practical use.

In order to overcome the overfitting problem in traditional ANN, a Bayesian approach, in which an *a priori* distribution of the parameters $\vec{w}$is used to regularize the training process. The prior probability distribution of $\vec{w}$,$p(\vec{w}|\vec{\alpha}$ is the regulation term that incorporates our prior belief of what constitute reasonable values of $\vec{w}$, where $\vec{\alpha}$ is a set of parameters that determine the distribution of the parameter $\vec{w}$, including both weights and biases; thus, it is known as a hyperparameter. Given a training dataset $D$ and the prior probability distribution of $\vec{w}$, one can obtain the posterior distribution of $\vec{w}$ using Bayes’ rule:

where$p(D|\vec{w})$is the true conditional probability of observing the training data $D$given a set of weight and bias parameters $\vec{w}$and is called the likelihood function for the data, and $p(\vec{w}|\vec{\alpha}$is the prior probability distribution of$\vec{w}$. The Bayesian training approach is to approximate the full posterior distribution of the network parameters rather than a maximum likelihood estimation using traditional ANN.

Gaussian approximation approach , which is using an evidence procedure in which the posterior density function of the network parameters $\vec{w}$ is locally approximated as being Gaussian and the posterior density function of the hyperparameters is assumed to be sharply peaked around the most probable values of the hyperparameters, was used for BANN implementation in this study.

An example, with the 4-5-1 (4 input features, 5 hidden units, and 1 output) BANN architecture, the output is calculated as following:

$$input\_row=\left[\begin{matrix} 1 & x_{1} & x_{2} & x_{3} & x_{4} \end{matrix}\right]$$

$$\text{ input\_to\_hidden (}first\_layer\_weights)=\left[\begin{matrix} \text{b}_{1}^{\text{(1)}} & \text{b}_{2}^{\text{(1)}} & \text{b}_{3}^{\text{(1)}} & \text{b}_{4}^{\text{(1)}} & \text{b}_{5}^{\text{(1)}} \\ \text{w}_{11}^{\text{(1)}} & \text{w}_{21}^{\text{(1)}} & \text{w}_{31}^{\text{(1)}} & \text{w}_{41}^{\text{(1)}} & \text{w}_{51}^{\text{(1)}} \\ \text{w}_{12}^{\text{(1)}} & \text{w}_{22}^{\text{(1)}} & \text{w}_{32}^{\text{(1)}} & \text{w}_{42}^{\text{(1)}} & \text{w}_{52}^{\text{(1)}} \\ \text{w}_{13}^{\text{(1)}} & \text{w}_{23}^{\text{(1)}} & \text{w}_{33}^{\text{(1)}} & \text{w}_{43}^{\text{(1)}} & \text{w}_{53}^{\text{(1)}} \\ \text{w}_{14}^{\text{(1)}} & \text{w}_{24}^{\text{(1)}} & \text{w}_{34}^{\text{(1)}} & \text{w}_{44}^{\text{(1)}} & \text{w}_{54}^{\text{(1)}} \end{matrix}\right]$$

$$\left[\begin{matrix} Z_{1} & Z_{2} & Z_{3} & Z_{4} & Z_{5} \end{matrix}\right]=\tanh(input\_row*input\_to\_hidden)$$

$$hidden\_values=\left[\begin{matrix} 1 & Z_{1} & Z_{2} & Z_{3} & Z_{4} & Z_{5} \end{matrix}\right]$$

$$\text{ hidden\_to\_output (}\sec ond\_layer\_weights)\text{ }=\left[\begin{matrix} \text{b}_{1}^{\text{(2)}} \\ \text{w}_{11}^{\text{(2)}} \\ \text{w}_{12}^{\text{(2)}} \\ \text{w}_{13}^{\text{(2)}} \\ \text{w}_{14}^{\text{(2)}} \\ \text{w}_{15}^{\text{(2)}} \end{matrix}\right]$$

$$output=\frac{1}{1+\exp(-hidden\_values*hidden\_to\_output)}$$

Weights (w) are available upon request (Contact: [m-giger@uchicago.edu](mailto:m-giger@uchicago.edu)).

**References, Appendices 1 and 2**

1. Huberty CJ: *Applied Discriminant Analysis.* John Wiley and Sons, Inc.; 1994.

2. Lachenbruch PA: *Discriminant Analysis.* New York: Hafner; 1975.

3. MacKay DJS: **Bayesian Methods for Adaptive Models, PhD Thesis.** California Institute of Technology, 1992.

4. Neal RM: *Bayesian learning for neural networks. Lecture notes in statistics.* New York: Springer-Verlag; 1996.

5. Bishop CM: *Neural Networks for Pattern Recognition.* Oxford, U.K.: Oxford University Press; 1995.

6. Nabney IT: *Netlab: Algorithms for Pattern Recognition.* London, U.K.: Springer; 2002.

7. Egan J: *Signal Detection Theory and ROC Analysis.* New York: Academic Press; 1975.

8. Kupinski MA, Edwards DC, Giger ML, Metz CE: **Ideal observer approximation using Bayesian classification neural networks.** *IEEE Trans Med Imaging* 2001, **20:**886-899.
