# Supplementary figures and images for: Relationships between computer-extracted mammographic texture pattern features and BRCA1/2mutation status: a cross-sectional study
Source: Breast Cancer Res. 2014 Aug 23;16:424. doi: 10.1186/s13058-014-0424-8 (PMC4268674; doi:10.1186/s13058-014-0424-8)

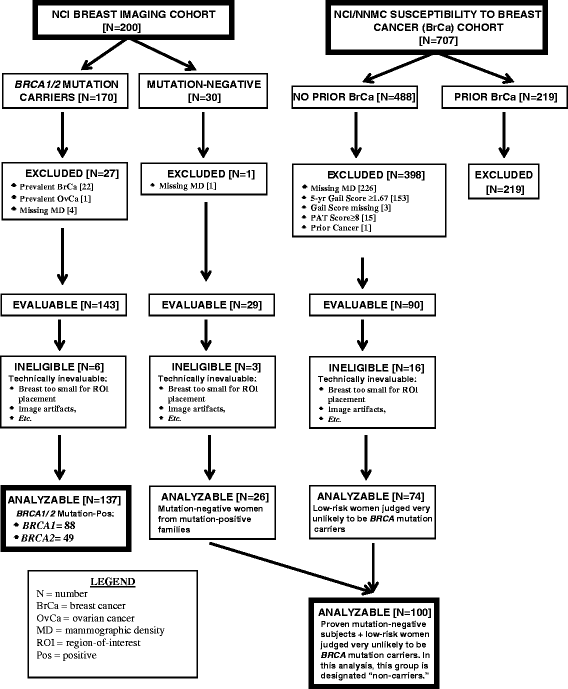

Supplement: Supplementary file 3 — Authors’ original file for figure 1 [file 13058_2014_424_MOESM3_ESM.gif]

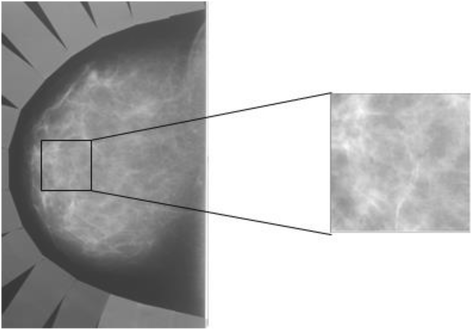

Supplement: Supplementary file 4 — Authors’ original file for figure 2 [file 13058_2014_424_MOESM4_ESM.gif]

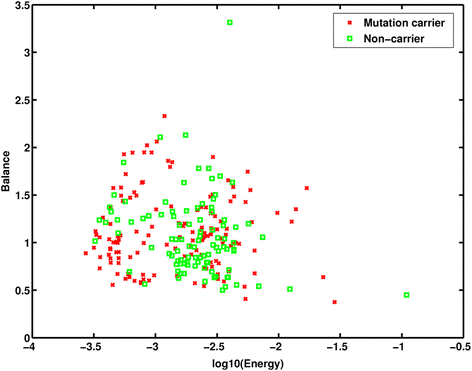

Supplement: Supplementary file 5 — Authors’ original file for figure 3 [file 13058_2014_424_MOESM5_ESM.gif]
